# Self-management challenges and psychosocial support needs in clean intermittent catheterization: a qualitative study of individuals with neurogenic bladder and caregivers

**DOI:** 10.3389/fresc.2026.1800715

**Published:** 2026-04-01

**Authors:** Lianyu Zou, Xiaomei Wu, Shun Chen, Shuluan Feng, Xiaohua Huang, Weiting Liu

**Affiliations:** 1 Department of Rehabilitation Medicine, Zhangzhou Affliated Hospital of FuJian Medical University, Zhangzhou, China; 2 The Second People’s Hospital Affiliated to Fujian University of Chinese Medicine, Fuzhou, China; 3 Fujian University of Traditional Chinese Medicine, Fuzhou, China; 4 School of Nursing and Midwifery, Edith Cowan University, Perth, WA, Australia; 5 JBI Affiliated Centre for Evidence Informed Nursing, Midwifery and Health Care Practice, Edith Cowan University, Perth, WA, Australia; 6 Research Centre of Prevention and Management for Chronic Disease, Edith Cowan University, Perth, WA, Australia

**Keywords:** clean intermittent catheterization, needs, neurogenic bladder, qualitative research, self-management

## Abstract

**Background:**

Clean intermittent catheterization (CIC) is widely regarded as the preferred approach to bladder management for individuals with neurogenic bladder (NB). Nevertheless, a considerable gap persists between evidence and clinical practice: patients and caregivers frequently encounter self-management barriers that compromise clinical outcomes during CIC. This qualitative study explores these barriers to provide an empirical foundation for developing tailored strategies that improve quality of care.

**Design:**

A descriptive qualitative study.

**Methods:**

From August to October 2025, a convenience sampling method was employed to recruit 16 participants individuals with NB or their long-term caregivers from the Department of Rehabilitation Medicine at Zhangzhou Affliated Hospital of FuJian Medical University, FuJian Province. Face-to-face semi-structured interviews were conducted, and thematic analysis was employed for data analysis.

**Results:**

Three themes and eight sub-themes were generated in this study: (1) Cognitive deficits and information dilemmas: barriers to health information delivery, a lack of personalized guidance, and cognitive biases and memory lapses; (2) Challenges in behavioral transformation: insufficient perception of risks and benefits and weakened self-management behaviors; (3) Need for an optimized health support system: greater access to psychological counseling, development of authoritative digital health education resources, and an integrated hospital-community management model.

**Conclusion:**

This study reveals the interconnected cognitive, behavioral, and psychosocial challenges that individuals with NB and their caregivers face in practicing CIC. Our findings underscore a critical need for interventions that address these specific implementation barriers. Future initiatives should focus on developing multidimensional, patient-empowering strategies that integrate personalized digital health education, ultimately to improve the quality and sustainability of CIC self-management.

**Impact:**

This study elucidates the complex practical and psychosocial challenges encountered by individuals with NB and their caregivers during CIC. Based on these findings, we propose a multi-pronged framework for improvement, targeting cognitive, behavioral, and systemic dimensions of care. This research lays a critical foundation for designing targeted, empowerment-based interventions and offers a comprehensive, system-level roadmap to bridge the persistent evidence-practice gap in CIC self-management.

**Reporting method:**

The research adheres to Consolidated criteria for reporting qualitative research (COREQ) guidelines.

## 1 Introduction

Neurogenic bladder (NB) is a bladder dysfunction resulting from the central or peripheral nervous systems, commonly observed in patients with spinal cord injuries, stroke, and multiple sclerosis (1). The condition presents a dual threat: it can lead to severe clinical complications, including recurrent urinary tract infections (UTIs), hydronephrosis, and renal failure, thereby compromising upper urinary tract function. Concurrently, its persistent storage and voiding symptoms profoundly impair patients’ quality of life across physiological, psychological, and social domains (2).

Intermittent catheterization (IC) is currently recognized internationally as the gold standard for bladder management in NB (1). Among IC methods, clean intermittent catheterization (CIC) is preferred for long-term self-management due to its procedural simplicity, cost-controllable, and suitability for home and community settings. Despite these advantages, adherence to CIC remains suboptimal, with study indicating that only about half of patients follow guideline recommendations (3). This discrepancy highlights a critical evidence–practice gap. To bridge this gap, we conducted a qualitative study to explore the practical challenges and psychsocial needs of individuals with NB and their caregivers. This study aims to elucidate the core determinants drive this gap and to estiablish an empirical foundation for developing empowerment-centered, multidimensional intervention that strengthen CIC implementation and long-term patient outcomes.

## 2 Background

### 2.1 Disease burden of neurogenic bladder

Normal micturition relies on the precise coordination of the central and peripheral nervous systems. NB is a form of bladder dysfunction resulting from damage to these neural pathways, leading to a loss of voluntary control over urination (1). Epidemiologically, NB is highly prevalent across various neurological conditions. For instance, it affects up to 80% of individuals with spinal cord injury (4), develops in approximately 75% of patients with multiple sclerosis (5), and is reported in 28%–79% of stroke survivors, with symptoms persisting in 15%-25% of this group one year post-event (6).

The pathophysiology of NB, often characterized by elevated intravesical pressure, reduced bladder compliance, and incomplete bladder emptying, NB significantly increases the risk of UTIs. Consequently, the incidence of UTIs can be as high as 54.9%, substantially higher than the 9.8% rate observed in the general population (7). These recurrent infections poses a considerable threat to the safety of the upper urinary tract and predisposes patients to long-term renal complications (8). Furthermore, the long-term management of NB imposes a substantial economic burden on both patients and society, with annual management costs estimated to range from $2,039.69 to $12,219.07 per patient (9).

### 2.2 Clinical value and application advantages of intermittent catheterization

IC, a bladder emptying technique for patients unable to void voluntarily, involves periodic and regular emptying of the bladder to mimic a near-physiological “storage-voiding” cycle. IC not only effectively reduces post-void residual urine volume and maintains bladder compliance but also reduces the risk of UTIs (10), protects upper urinary tract (renal) function, and prevents renal failure (11). Furthermore, emerging evidence suggests that IC also plays a significant role recovering autonomous bladder control and improving long-term clinical outcomes (4).

From a health economics perspective, IC demonstrates superior cost-effectiveness ratio compared to long-term indwelling catheterization. By reducing complications such as UTIs and upper urinary tract damage, IC decreases the need for rehospitalization and additional treatments, thereby offering significant advantages in lowering overall medical costs and societal burden (12). Consequently, IC is recommended by the International Continence Society as the safest first-line measure for bladder emptying in individuals with NB and is considered the gold standard for managing NB dysfunction (1).

Based on aseptic technique, IC can be categorized into CIC and sterile intermittent catheterization. Studies have reported no significant differences between these two methods in terms of reducing UTI incidence, decreasing residual urine volume, or improving bladder capacity (13). However, CIC is recommended as the preferred bladder management strategy in non-emergency setting (14), and is also the bladder management method most frequently chosen by spinal cord injury patients at discharge (15). This preference is attributed to its operational simplicity, lower costs for supplies and treatment, and ease of implementation in community and home settings, facilitating patient self-management.

### 2.3 Current application status of CIC in individuals with NB

Despite its well-established clinical value, achieving standardized implementation of and long-term adherence to CIC remains a significant challenges in clinical practice. These challenges are multifaceted. For instance, inconsistencies in the professional competency of clinical nurses, the primary educators for CIC, can hinder effective patient training (16). Furthermore, existing educational approaches are often standardized, failing to address the individualized needs of patients (17). A paricicularly prominent issue is poor adherence, whice persists across the continuum of care, from hospitalization to discharge. During hospitalization, up to 50.2% of patients fail to adhere to guideline-recommended CIC practices, and these patients exhibit a higher UTI incidece compared to their adherent counterparts. After discharge, only 15% of patients fully adhere to the CIC technique (18). Collectively, these challenges highlight a significant evidence-practice gap and underscore the urgent need to understand its underlying causes to improve the translation of evidence into routine care.

While extensive quantitative research has focused on catheter materials, clinical efficacy, and complication management, the lived experiences and psychosocial needs of the end-users—patients and their caregivers—have been largely overlooked. Consequently, the root causes of the evidence-practice knowledge gap remain poorly understood. To address this, the present study will employs a qualitative research approach to explore the management challenges and psychosocial needs needs of individuals performing CIC. The findings are intended to provide an empirical foundation for developing targeted patient empowerment interventions designed to bridge this gap.

## 3 The study

### 3.1 Aims

The primary objective of this study was to investigate the practical challenges and psychosocial support needs experienced by by individuals with NB or their caregivers during the implementation of CIC. Concurrently, it sought to explore targeted management strategies, aiming to provide an empirical basis for bridging the “evidence-practice” gap. To achieve these aims, this qualitative inquiry was guided by three central domains of investigation:

1. Participants’ understanding of CIC and the specific strategies employed in its daily management.
2. Perceived challenges and difficulties encountered during the long-term implementation of CIC.
3. Unmet needs and desired future support concerning CIC guidance and education.

## 4 Methods

### 4.1 Design and theoretical framework

This study is theoretically grounded in the constructivist paradigm. This perspective posits that reality is not a single objective entity but is actively constructed and subjectively interpreted by individuals within dynamic socio-cultural contexts, thereby exhibiting multiplicity and subjectivity (19). Guided by this theoretical perspective, a qualitative descriptive research design was adopted. This methodology was specifically chosen to gain an in-depth understanding of participants’ lived experiences and subjective perceptive during the CIC management process within their unique life circumstances. This design aligns closely with the constructivist framework by enabling a thorough untangling of the meanings that individuals with NB and their long-term caregivers construct, thereby effectively addressing the core objectives of the study. To ensure methodological rigor and transparency, the investigator strictly adhered to the Standards for Consolidated criteria for reporting qualitative research (COREQ) (20).

### 4.2 Study setting and recruitment

This study was conducted in the Department of Rehabilitation Medicine at Zhangzhou Affliated Hospital of FuJian Medical University, FuJian Province, China. A convenience sampling method was employed to recruit participants. Eligible individuals with NB or their long-term caregivers were recruited at the aforementioned medical institution between August to October 2025. During the initial recruitment phase, the investigator identified and approached potential participants who met the inclusion criteria. A detailed research information sheet was distributed, and the investigator provided detailed explanations regarding the study purpose, procedures, potential risks, and benefits. Potential participants were explicitly stated that participation was stictly voluntary, that they retained the right to withdraw at any time without penalty, and that their routine treatment plans would remain unaffected. Following an adequate period for consideration, individuals who expressed interest contacted the investigator by telephone or in person. For all interested candidates, the study details and expectation for cooperation were reiterated using the standardized informed consent form. Only after written informed consent was formally obtained were participants officially enrolled in the study.

### 4.3 Inclusion and exclusion criteria

The study included adult individulas (aged ≥18 years) with NB, or their long-term caregivers, whose diagnosis had been independently confirmed by senior physicians with advanced professional titles. The specific inclusion criteria for participants were:

1. Patients currently undergoing CIC procedures, or their long-term caregivers (including immediate family members and spouses).
2. Individuals who voluntarily agreed to participate in the interview and provided written informed consent.

Participants were excluded if they met any of the following criteria:

1. Paid caregivers (e.g., hired nursing assistants or domestic helpers).
2. Individuals with language communication barriers who were unable to participate in interviews primarily refer to those with congenital aphasia and stroke-related aphasia.
3. Individuals with a history of psychiatric disorders that might affect the validity of the interview data.

Given the qualitative nature of this study, the final sample size was determined based on the principle of thematic saturation. Thematic saturation was defined as the point at which no new themes or significant properties of existing themes emerged from the interview data (21, 22). Following preliminary interviews with 5 NB patients and 9 long-term caregivers of NB patients, data saturation was achieved for both groups. To test the robustness of saturation, two additional participants were recruited. As no new themes emerged, recruitment was terminated. A total of 16 participants were ultimately included in the study.

### 4.4 Data collection

Upon receiving signed informed consent, interviews were scheduled at a time convenient for the participants, ensuring no conflict with their rehabilitation therapy schedules. All interviews were conducted in private, quiet rooms to maintain confidentiality and minimize distractions. All interviews were conducted by two registered nurses (Shuluan Feng and Xiaohua Huang), both of whom held bachelor's degrees. The interviews possessed extensive clinical knowledge of rehabilitation nursing and had received formal training in qualitative interview techniques. Prior to commencing the interview, the researchers established rapport with each participant to foster an open and trusting environment. Subsequently, general demographic data were collected. Followeing this, the face-to-face semi-structured was conducted. The semi-structured interview guide was developed based on the research objectives and a review of relevant literature (23). To ensure content validity and applicability, the guide was first reviewed by a senior clinical nursing expert. After that, the guide was pilot-tested with two participants to assess the clarity, flow, and appropriateness of the questions. Based on the feedback from the expert consultation and the pilot participants, the guide was revised and refined. The final interviews guide comprise six primary items (Table 1). Thoughtout the formal interview process, the interviewer maintained a neutral stance, avoided leading questions. Techniques such as probing, clarification, and paraphrasing were employed to to elaborate on their experiences and to clarify ambiguous statements. Additionally, valuable content was further explored through in-depth questioning. The interviewer also documented non-verbal cues (e.g., tone, intonation, facial expressions) to capture potential underlying information. Each interview lasted approximately 20–30 min.

**Table 1:** Semistructured interview guide.

Number	Interview questions
Question 1	Are you aware of the benefits of CIC for bladder management?
Question 2	Are you familiar with the concept of bladder safe capacity, and to what extent do you understand the purpose of measuring it?
Question 3	Do you know the appropriate timing for performing CIC on patients, and how do you carry it out on a daily basis?
Question 4	Are you aware that fluid intake control is necessary when implementing CIC, and how do you manage this?
Question5	What difficulties have you encountered during the catheterization process, and how were they resolved?
Question6	In which areas would you like to receive further assistance or guidance from healthcare professionals in the future?

### 4.5 Data analysis

Demographic data were analyzed using SPSS Statistics 26.0 (IBM Corp., Armonk, NY, USA). Continuous variables were expressed as mean ± standard deviation (SD), while categorical variables were presented as frequencies (n) and percentages (%). All interview were audio-recorded and transcribed verbatim within 48 h. To ensure accuracy and fidelity, two researchers (Shuluan Feng and Xiaohua Huang) independently transcribed the recordings, annotating non-verbal information. The transcripts were subsequently verified and refined by the original interviewers in consultation with the respondents.

The verified textual data were analyed using the six-step thematic analysis framework developed by Braun and Clarke (24). Two researchers (Lianyu Zou and Shun Chen), both proficient in qualitative research analysis methods, independently coded the data with the assistance of NVivo software and WPS Excel. The analysis proceeded through the six defined stage: (1) familiarization with the data; (2) generating initial codes; (3) searching for themes; (4) reviewing themes; (5) defining and naming themes; and (6) producing the report. Following independent analysis, the two researchers met to cross-compared their coding result. Any discrepancies were resolved through discussion and consensus. A third researcher (Weiting Liu) was consulted to arbitrate any unresolved disagreements, ensuring the reliability of the final thematic structure.

### 4.6 Rigour

To ensure the rigor of this study, the core principles of credibility, transferability, dependability, and confirmability were strictly adhered to. The specific strategies implemented were as follows: Regarding credibility, researchers first established rapport with participants (patients or long-term caregivers) by engaging in per-interview discussions about their medical or care experiences. This process fostered trust, facilitating the acquisition of more authentic and in-depth information. Additionally, researcher triangulation strategy was employed, involved independent data analysis and cross-verification of the results by two researchers. Preliminary findings were also returned to the participants for member checking to ensure that the interpretations accurately reflected their genuine experiences.In terms of transferability, the study provides a “thick description” of the research context, clearly delineated the inclusion criteria, sampling methods, socio-demographic characteristics of the participants. For dependability, a comprehensive audit trail was maintained, including signed informed consent forms, interview audio recordings and notes, verbatim transcripts, and all documentation coding levels and themes generated. All original materials were properly archived. To ensure confirmability, the transcription of audio recordings was conducted independently by two researchers, followed by cross-comparison of the transcripts. Any discrepancies were resolved by jointly reviewing the recordings until consensus was reached. The verified transcripts were then returned to the respondents for content confirmation, yield a reliable final transcript. During the data analysis phase, dual independent coding and theme extraction were performed, followed by cross-checking. Any disagreements were resolved by consulting a third researcher to minimize analytical bias.

### 4.7 Research team and reflexivity

This study was conducted by an interdisciplinary team with expertise in both qualitative methodology and clinical practice. The core team included the first author (Lianyu Zou), a master's student with three years of practical experience in rehabilitation nursing and specialiazed training in qualitative research; Two doctoral candidates from Australia and China; and three clinical rehabilitation nursing specialists. The interviews were conducted by two experienced nursing professionals (Shuluan Feng and Xiaohua Huang). Their solid clinical expertise was crucial for establishing rapport and facilitating an in-depth understanding of the participants’ expressions and internal experiences. To ensure the quality of the interview, the first author (Lianyu Zou) provided specialized qualitative interview training and assessment to the interview prior to the data collection phase, ensuring they acquired fundamental qualitative interviewing skills. The overall methodological rigor of the research was supervised by a doctoral candidate (Weiting Liu) proficient in qualitative research methods. The team fully acknowledged the potential influence of their subjective positions and backgrounds on the study. Therefore, reflective practice was consistently implemented throughout the research process. Therefore, reflexivity was a cornerstone of the study. Reflective practice was consistently implemented throughout all stages, including study design, data collection, analysis, and manuscript development, and regular team discussions were held to cross-validate findings. All conclusions were drawn with the aim of faithfully representing the participants’ psychological experiences and internal perspectives, minimizing analytical bias stemming from the researchers’ pre-understandings.

## 5 Results

### 5.1 Characteristics of participants

A total of 16 participants were enrolled (5 patients and 11 long-term caregivers). The mean was 45.94 ± 12.06 years, ranging from 22 to 60 years. the sample was predominantly male. Key clinical characteristics are summarized as follows: ①Most participants had undergone only one session of CIC education, primarily conducted by nurses. ②The majority of participants had been performing CIC for ≤ 3 months, with only 2 participants having continued CIC for more than one year (See Table 2 for full details).

**Table 2:** Summary of characteristics of informants.

Characteristics	Mean (SD) or frequencies
Age (years)	45.94（12.06）
Gender: *n* (%)	
Male	11 (68.75%)
Female	5 (31.25%)
Degree of education: *n* (%)	
Primary education	5 (31.25%)
Secondary education	7 (43.75%)
Higher educaiton	4 (25.00%)
The relationship between the participants and the patients: *n* (%)	
Self	5 (31.25%)
Couple	5 (45.45%)
Children	4 (36.36%)
Brothers	1 (9.09%)
Grandpa	1 (9.09%)
Participants’ occupations: *n* (%)	
Agricultural worker	4 (25.00%)
Service industry worker	5 (31.25%)
Manufacturing industry worker	3 (18.75%)
On the drift	4 (25.00%)
Number of times receiving CIC teaching guidance: *n* (%)	
1 times	9 (56.25%)
2–5 times	6 (37.50%)
6–10 times	1 (6.25%)
Teaching staff: *n* (%)	
Doctor	1 (6.25%)
Nurse	12 (75.00%)
Nursing workers	2 (12.50%)
Nurses and caregivers	1 (6.25%)
CIC duration: *n* (%)	
<1 months	5 (31.25%)
1–3 months	8 (50.00%)
4–6 months	1 (16.25%)
>6 months	2 (12.50%)

### 5.2 Themes

Qualitative analysis of the interview data revealed three major categories encompassing eight distinct subthemes related to the challenges in CIC management and psychosocial service needs of individual with NB or their long-term caregivers. These thematic findings are comprehensively detailed in Table 3.

**Table 3:** Summary of categories and subcategories.

Themes	Category	Subcategory
1	Cognitive Deficits and Information Dilemmas	Barriers to Health Information Delivery
		Lack of Personalized Guidance
		Cognitive Biases and Memory Lapses
2	Challenges in Behavioral Transformation	Insufficient Perception of Risks and Benefits
		Weakened Self-Management Behaviors
3	Need for an Optimized Health Support System	Greater Access to Psychological Counseling
		Development of Authoritative Digital Health Education Resources
		Establishment of a Hospital-Community Linkage Management Model

#### 5.2.1 Theme 1: cognitive deficits and information dilemmas

##### 5.2.1.1 Sub-theme 1:barriers to health information delivery

Barriers to health information delivery during CIC implementation represent a significant contributor to the evidence-practice gap. The interview findings revealed two primary modifiable factors responsible for these barriers.

First, the health education provided by healthcare professionals during CIC implementation often lacked systematicity. Consequently, the vast majority of interviewees demonstrated significant knowledge regarding of fundamental concepts. For instance, paticipants commonly lacked awareness of the “maximum bladder safe capacity” and misunderstood core health information, including appropriate catheterization frequency, fluid intake management, and the purpose of bladder function tests. This knowledge deficit directly hindered the effective implementation of the evidence-based practics.

“(With a puzzled expression) What is safe capacity? Do we need to measure it? What do we need to do?” (P6)

“Previously, it was mentioned that fluid intake should be strictly controlled. I would like to ask whether the juice, soup, and nutritional supplements consumed with each meal count towards the total fluid intake? Since we have good digestion, it should be fine to feed more soup and juice, right?” (P3)

Second, variable professional competencies among health educators result in ambiguous or unclean information delivery. This further impeded the ability of individuals with CIC and their long-term caregivers to perform standardized procedures. This issue was compounded by the frequent rotation of nursing staff, whice led to inconsistencies in the health education content delivered. Participants reported receiving conflicting advice from different nurses, making it difficult to obtain unified operational guidance. Notable, some interviewees reported acquiring CIC skills from nursing aides rather than professional healthcare providers. These findings underscore the challenges in standardizing training protocols and maintaining consistent professional competence among educators.

“The nurses change frequently. Sometimes when we ask questions, the answers are vague, and we are left confused—for instance, about how much water to drink or how often to catheterize.” (P11)

“After the nursing aide left, I performed the catheterization myself. I didn't ask a nurse to observe. I just followed the steps taught by the aide, assuming it would be correct.” (P10)

##### 5.2.1.2 Sub-theme 2:lack of personalized guidance

Effectively translating health-related theoretical knowledge into standardized CIC management practices represents a core challenge for patients and their long-term caregivers. Most interviewees reported encountering numerous specific difficulties during actual CIC procedures, highlighting an urgent need for targeted professional guidance. These issues were primarily manifested in the following aspects:

First, unfamiliarity with the anatomical structure of the urinary system led to technical difficulties during catheterization. More concerningly, when faced with such operational challenges, individuals and caregivers often resorted to inappropriate self-management strategies, thereby posing potential safety risks.

“In the beginning, since there are several openings, I frequently inserted the catheter into the wrong one. I realized it was wrong when no urine drained.” (P7, P12)

“When I initially inserted it into the wrong location, I would withdraw the catheter and reinsert it. If insertion was still difficult, I would apply more lubricant using the lubricating bulb, which then allowed successful insertion.” (P2, P7)

Furthermore, the establishment of an effective continuous follow-up mechanism was critical for individuals and caregivers to maintain standardized CIC management practices. The absence of professional guidance or delayed feedback and corrections significantly undermine implementation outcomes. Participants expressed confusion regarding ongoing management strategies, underscoring their need for professional guidance and timely feedback to adjust care plans.

“The doctor didn't say I could stop, so I’ve been persistently performing catheterization…” (P2)

“I seem to lack understanding about fluid intake—for instance, when there's a lot of urine these past couple of days, how should I adjust her intake appropriately?” (P4)

Although infection is a commonly perceived risk, some interviewees mentioned their limited understanding of comprehensive infection prevention and control measures. A few even demonstrated a notable disconnect between theoretical knowledge and practical application, highlighting the need for healthcare professionals to emphasize guidance on infection control content in CIC education.

“Later, they (the nurses) explained it twice. At the beginning, I still couldn't remember clearly and didn't fully understand what should not be touched.” (P13)

Moreover, individuals and caregivers exhibited limited coping capacity when managing specific situations, such as complication recognition and management during menstruation. This specific finding underscores an urgent necessity for healthcare professionals to strengthen health education regarding coping strategies in these areas.

“During menstruation, catheter care becomes more challenging. There's a risk of menstrual blood contaminating the catheter, which seems more likely to cause infection. Yet, this situation recurs monthly, making it quite troublesome.” (P12)

“My mother suddenly developed a fever recently……Could the fever be related to the catheter……. In such a case, does the catheter need to be reinserted?” (P16)

##### 5.2.1.3 Sub-theme 3:cognitive biases and memory lapses

The interviews revealed that some participants held cognitive biases regarding CIC self-management due to an incomplete understanding of the disease. These misconceptions included beliefs such as, “the more fluid intake, the better” and “regulating residual urine volume by controlling fluid intake”. Such biases resulted in failure to adhere to evidence-based, fixed hydration schedules and led to arbitrary management practices. Cognitive limitations concerning hydration can, on one hand, impair the ability to judge the appropriateness of one's own behaviors, and on the other hand, lead to unstable bladder capacity, thereby increasing the risk of urinary retention and upper urinary tract injury.

“I do it arbitrarily. Rehabilitation sessions have fixed schedules. If there are no sessions, I feed her a few more syringes when I have time, and fewer when I'm busy—no strict control. Isn't more water always better?” (P2)

“If the residual urine volume is high, I reduce my water intake, but I still catheterize twice a day as usual.” (P5)

Some interviewees reported omitted key practical information or failing to follow medical instructions correctly when implementing intermittent catheterization self-management strategies. This was often attributed to memory decline or blurred recollection, whice subsequently affected the recovery of bladder function. The procedural complexity of CIC was significantly compounded by these cognitive impairments.

“My mind isn't working well—I often forget the steps. When I catheterize, either I don't have all the supplies ready, or after putting on gloves, I remember I still need to unpack the cotton balls and catheter. It just feels rushed and messy.” (P6)

#### 5.2.2 Theme 2: challenges in behavioral transformation

##### 5.2.2.1 Sub-theme 1: insufficient perception of risks and benefits

A considerable number of interviewees demonstrated insufficient knowledge regarding the therapeutic rationale and long-term benefits of CIC for bladder management. This deficit led to the procedure being performed passively, often translating into a lack of initiative to seek out relevant information and subjectively neglect of consistent management practices. Concurrently, participants exhibited a limited awareness of the potential harms associated with non-adherent behaviors. This overall deficient perception of both risks and benefits emerged as a significant factor constraining the effectiveness and maintenance of CIC management.

“The nurse briefly explained the purpose of intermittent catheterization when she first taught me, but I didn't pay much attention. Now that we are in the hospital.. if I don't understand something, I ask you doctors and nurses. I haven't really concerned myself with what the specific benefits are.” (P3)

“Sometimes if I drink more water or extend the time between catheterizations, the volume occasionally exceeds the maximum safe bladder capacity. An occasional exceedance probably doesn't cause significant harm to the body, right?” (P7)

##### 5.2.2.2 Sub-theme 2:weakened self-management behaviors

From a behavioral perspective, participants generally exhibited low adherence to CIC self-management protocol, manifesting as deficient self-management behaviors. This was primarily reflected in inconsistent (or non-systematic) fluid intake control and a long-term omission of essential bladder function monitoring, such as tracking post-catheterization urine volumes.

“I just control the total daily amount to around 2000mL; I haven't been meticulous about controlling the fluid intake for each meal.” (P13)

“I don't measure it anymore (the volume of urine drained), I just empty it directly into the toilet.” (P12)

Concurrently, the interviews revealed a significant propensity for over-reliance on healthcare professionals or nursing aides. This reliance was coupled with a diminished awareness of the need for personal participation and operational oversight. This delegation of responsibility consequently resulted in a “practice gap”, wherein participants (or their long-term caregivers) were not actively engaged in the decision-making or verification processes of the CIC procedure.

“Since there is a nursing aide now, she often helps with the catheterization. I ……letting the nursing aide handle it directly; I don't manage her (the nursing aide).” (P16)

#### 5.2.3 Theme 3: need for an optimized health support system

##### 5.2.3.1 Sub-theme 1: greater access to psychological counseling

Neurogenic bladder, as a chronic disorder resulting from neurological impairment, is characterized by a prolonged process of physiological functional reconstruction. The slow therapeutic progress and persistent uncertainty regarding treatment outcomes engender negative significant emotions distress in both patients and caregivers. This psychological burden often compromises adherence to self-management protocols and undermines trust in the overall treatment trajectory. Several interviewees explicitly voiced considerable anxiety concerning the uncertain prospects and the necessity of indefinite catheterization.

“I am concerned about how long this catheterization will be necessary…… When will this condition improve? Or will she have to continue catheterization indefinitely? Will she ever recover fully and be able to urinate independently?” (P2)

It is also noteworthy that CIC is an intimate procedure involving privacy concerns, presents significant psychosocial constraints that even extend beyond the physical realm. The required management and long-term reliance on CIC for maintaining urinary function impose considerable social pressures and inconvenience. This results in a strong expressed desire for a return to social normalcy and integration:

“Living with this condition indefinitely, I don't know when I can return to a normal life. Sometimes when I go out with friends, I feel somewhat awkward—it's really inconvenient.” (P5)

##### 5.2.3.2 Sub-theme 2: development of authoritative digital health education resources

The ubiquitous nature of online health information offers individuals convenience but simultaneously poses challenges regarding information quality and discernment. In this complex digital environment, participants frequently expressed a strong prefrence for acquiring reliable and professional health knowledge directly from medical institutions. Therefore, it is necessary for medical institutions to establish diversified information access channels to meet the knowledge needs of various individuals groups.

“Regarding the problem of difficult catheterization, I have also sought some information online, but the methods found on the internet lack guaranteed authority. I prefer to ask you (healthcare staff) and resolve my concerns through you.” (P1)

Forthemore, clinical practice, it has been observed that the delivery of a large volume of health information within a short period places high demands on the learning capacity of individuals with NB and their caregivers, often leading to memory lapses or sub-optimal learning outcomes. In response to this issue, some interviewees mentioned that during CIC training and guidance, they hoped to receive paper-based materials or electronic information channels to achieve complementary multi-modal health education resources.

“Is there any way for us to access information about the hydration plan and catheterization frequency, for example, through official public accounts or videos…… Having a way to review the information repeatedly would help us family members perform better.” (P11)

“Regarding the hydration schedule, could an electronic version be provided? Paper versions are easily lost. If there's an electronic copy, I could refer back to it if I forget. ……” (P14)

##### 5.2.3.3 Sub-theme 3: establishment of a hospital-community linkage management model

The absence of a robust regional medical collaboration mechanism severely constrains the accessibility and convenience of comprehensive healthcare services. Interview results consistently revealed that establishing a collaborative management model between the primary treating hospital and the responsible community medical institutions has emerged as a common and urgent demand among individuals and their caregivers. This finding underscores the necessity of integrated care networks to bridge current systemic gaps in care delivery.

“Sometimes when symptoms occur (such as cloudy urine or urinary discomfort)……The community health center is very close to my home. If there were collaboration between the community center and your hospital, it would be very convenient for me to go there directly for assistance.” (P1)

## 6 Discussion

This study evaluated the knowledge of CIC among individuals with NB or their long-term caregivers, identified the challenges in during CIC practice, and explored potential solutions. The overarching goal was to provide evidence-based insights for healthcare institutions to bridge the “evidence-practice” gap in CIC. This findings culminated in three principal discussion themes: 1) addressing health literacy deficits through optimized educational pathways; 2) overcoming barriers to behavioral adherence and empowering patient self-management; 3) recognizing diverse service needs to foster the integration and optimization of health support systems.

### 6.1 Addressing health literacy deficits and optimizing educational pathways

From the perspective of health educators, our finding highlight significant practical challenges in CIC management, primarily stemming form inconsistent professional competence and a lack of systematic educational implementation. This situation widens the “evidence-practice” gap. Our result align with recent studies (25–27) indicating that while over 90% of clinical nurses report a high demand advanced NB or CIC training, inconsistencies in existing CIC practice guidelines (28, 29) compound this issue, impeding improvement in clinical practice quality. These findings underscore the need for several interventions: 1) Future research must focus on generating higher-level evidence to guide practice. 2) At the departmental level, we recommend establishing specialized CIC management team with verified professional and teaching capabilities to lead translational evidence-based training, ensuring the consistency, accuracy, and comprehensiveness of information. 3) To address non-systematic education, a refined CIC health education checklist (30) could be develop to standardization and improve the completeness of nurse instruction. 4) healthcare institutions should leverage the recent Chinese group standard for adult intermittent catheterization (October 2024) to enhance staff proficiency through dedicated online and offline traning.

From the perspective of patients and caregivers, this study identified a strong, unmet demand for continuous follow-up and personalized guidance. We found that conventional, didactic (“cramming-style”) health education is often insufficient, leading to suboptimal learning outcomes. Therefore, future CIC teaching practices must evolve. Based on the needs identified in this study, we suggest that clinical professionals utilize anatomical models for concrete explanations and prioritize in-depth teaching on common CIC difficulties and their management, including emergency guidance for catheterization difficulties, to mitigate medical risks. Furthermore, this study reveal that patients and caregivers generally demonstrate deficient capacity for complication identification and management, a finding consistent with prior research (14, 31). This highlights the urgent need to strengthen complication-focused education. for patients and caregivers is a key direction for future health education. Crucially, CIC is a dynamic process. The strong need for dynamic follow-up guidance suggests that robust inter-shift handover mechanism must be rigorously implemented to ensure timely patient behavior follow-up, allowing for prompt corrective guidance and integrating following-up throughout the entire CIC management process.

Additionally, our study identified deficits in knowledge transfer and retention among some patients and caregivers, potentially linked to factors such as educational background or memory decline. This observation necessitates a more tailored educational approach. We recommend that future CIC teaching practices include a thorough assessment of patient and caregiver educational levels and cultural beliefs to personalize instruction. Standardized educational materials, such as pamphlets on fluid intake plans or instructional videos, should be utilized to understand and reinforce key information. Concurrently, the “Teach-back” method (32) should be introduced to regularly assess comprehension of CIC health knowledge. This approach targets specific weak areas for reinforcement, establishing a closed-loop education model of “education-feedback-re-education” to continuously solidify patients and caregivers self-management capabilities.

### 6.2 Overcoming the challenges in behavioral practice transformation and empowering self-management

This study found that the weakening of patient and caregiver self-management behaviors is primarily manifested in two dimensions: low adherence and excessive dependence. This finding is consistent with the literature, notably a large-scale clinical study by He M et al. (3), which revealed that up to 50.2% of CIC procedures do not align with guideline recommendations, thus directly reflecting the “evidence-practice” gap in CIC management. Further research (33) indicates that while most patients and caregivers initially master CIC operational skills, adherence tends to decline over time. Adherence is a core metric that must be considered when formulating effective CIC health education measures (31). As reported by several interviewees, the confict between the prescribed CIC schedule or fluid intake plan and their rehabilitation therapies reduce their adherence. In address to this issue, we strongly recommended that clinical healthcare professionals adopt a shared decision-making model (14) when developing CIC management plans. By fully incorporating the patient's rehabilitation and lifestyle needs, a feasible, individualized management plan can be collaboratively developed with patients and caregivers to proactively eliminate these barriers and enhance intrinsic motivation.

Notably, patients with stroke or spinal cord injuries and their caregivers exhibited an excessive reliance on nurses or nursing aides during CIC management. This pattern of weakened self-management aligns with findings from cross-sectional surveys (34). Deeper analysis suggests that these dependent or low-adherence behaviors may stem from vague perception of the benefits of CIC and the associated complication risks. This lack of risk-benefit clarity manifesting as an attitude of “indifference” or “unconcern,” which severely undermines the motivation required for autonomous management. This conclusion is further supported by the work of Zhang et al. (35).

Focusing on strategies to improve self-management, future research should explore the development of a structured prompt list for CIC self-management issues (36). This resource would provide a systematic framework for patients and caregivers, effectively boosting their confidence and capability.

Furthermore, in the management of CIC for individuals with NB, clinical departments clinical departments may consider implementing regular online follow-up services delivered by specialized nurses,and leveraging digital health tools, such as WeChat mini-programs (37), can enable the targeted delivery of personalized information, CIC-related knowledge, and risk-specific education. Such an approach holds promise for reinforcing patients’ and caregivers’ capacity for self-management, thereby helping to bridge the gap between behavioral knowledge and sustained clinical practice.

### 6.3 Clarifying diversified service needs and integrating an optimized health support system

The interviews revealed that individuals with NB and their caregivers face multifaceted support needs during the self-management process, with psychological stress, information acquisition, and educational formats being particularly prominent.

Firstly, psychological stress arises from uncertain treatment outcomes and social distress, which is consistent with the conclusions of several previous studies (2, 38). This suggests that clinical practice should prioritize the integration of psychological counseling into routine management. Departments should consider organizing patient support groups, leveraging peer support to alleviate psychological anxiety and distress. This approach may, in turn, enhance self-management capabilities and treatment adherence.

Secondly, regarding information acquisition, patients and caregivers exhibit a strong reliance on authoritative information provided by healthcare professionals, a finding that aligns with the research results of Fitzpatrick MA et al. (39). However, a significant barrier exists: although guidelines for NB management are available, recommendations for nursing practice remain ununified across different publications (40). Therefore, healthcare institutions should continue to conduct high-quality, evidence-based research to refine and standardize relevant guidelines, providing precise and reliable foundations for NB nursing practice. As hospitals are the most trusted information source, they should also optimize information delivery methods to ensure patients can access more scientific and reliable educational information.

Third, interviewees showed diverse preferences for educational formats, weighing the benefits of electronic vs. paper-based materials.This mirrors findings by Fitzpatrick MA et al. (41), who noted that some patients prefer not only richly illustrated materials but also peer-led health education models. Furthermore, video-assisted NB training has been shown to effectively enhance patients’ practical skills and confidence (42). Consequently, traditional health education formats, such as verbal explanations and paper materials, are no longer sufficient to meet the diverse educational demands of patients and caregivers. Future efforts in NB nursing management should focus on optimizing and enriching health education resources, promoting a combined “verbal-paper-electronic” health education model to comprehensively improve overal educational effectiveness.

Finally, NB resulting from spinal cord injury often requires long-term management, yet readiness for hospital discharge is low (43), and a strong need for post-discharge informational support (44). Effective social support systems have been proven to play a significant role in enhancing self-management capabilities (35). Therefore, we recommended that well-resourced tertiary hospitals take a leading role in the promotion and training of NB nursing techniques. This leadership should aim to optimize the allocation of regional medical resources, ensure the convenience patient access to care, and alleviate the medical burden on higher-level hospitals. This strategy promotes the implementation of a hospital-community linkage management model, whice is essential for improving patients’ long-term self-management abilities and quality of life.

### 6.4 Strengths and limitations of the work

This study focuses on bladder management in adult NB and, for the first time, analyzes the self-management challenges of CIC among adult individuals with NB and the service needs of CIC patients or caregivers regarding CIC education and guidance from their perspectives. The findings contribute to a better understanding of the CIC experience in individuals with NB in subsequent research or clinical practice and facilitate the development of feasible future CIC-based bladder management strategies. This may improve the quality of CIC bladder management in individuals with NB and provide robust empirical evidence to bridge the “evidence-practice” gap. The study's findings must be interpreted in the context of several limitations. The use of convenience sampling and a single-center design (recruiting from one tertiary hospital in China) may introduce selection bias. This inherently limits the generalizability of our findings. Consequently, whether the identified management challenges and psychological needs are consistent across different healthcare settings or cultural contexts requires further investigation. To partially mitigate this limitation, we employed a purposeful recruitment strategy, deliberately seeking participants with diverse ages, genders, educational backgrounds, and disease etiologies. This approach was intended to enhance the sample's representativeness and the richness of the qualitative data.

### 6.5 Recommendations for future research

This study illuminates the specific self-management challenges and multidimensional unmet needs faced by individuals with neurogenic bladder (NB) and their caregivers. Building on these findings, future research should focus on developing and validating novel intervention models. We specifically recommend investigation into: 1) Innovative Management Strategies: Exploring the efficacy of digital health education platforms and mobile health applications to provide accessible, continuous support. 2) Empowerment Centered Programs: Based on evidence to further explore innovative multidimensional interventions centered on patient “empowerment.” The objective of these initiatives should be to evolve clinical education from a unidirectional, static model of skill transfer toward a dynamic, empowering, and continuous health education pathway. Furthermore, future qualitative research should incorporate multiple perspectives (e.g., from physicians, nurses, and healthcare leaders) to more comprehensively explore the determinants of the evidence-practice gap. Such an approach would more comprehensively elucidate the sources of the evidence-practice gap and provide the multifaceted understanding needed to address it.

### 6.6 Implications for clinical practice

While research on intermittent catheterization in NB has prioritied technological advances, the clinical challenges and the psychological experiences of patients or caregivers performing CIC have received limited attention. To date, evidence addressing these practical and psychological aspects remains scarce. This qualitative study was therefore designed to investigate these unresolved issues. The findings illuminate the management challenges and psychosocial service needs faced by this population. In response, this study explores potential improvements across the cognition, behavior, and support systems dimensions, proposing systematic solutions to bridge the “evidence-practice” gap (Table 4).

**Table 4:** Implications for CIC implementation.

Content	Implications for Policy	Implications for Practice	Implications for Education
Identifying Deficiencies in Health Cognition and Optimizing Educational Practice Pathways		√	
Develop a comprehensive and refined health education checklist for CIC.		√	√
Clinical departments should establish a dedicated CIC management team with sufficient professional knowledge and teaching capabilities.	√	√	
Actively organize online and offline training sessions and exchange meetings to enhance the professional competence of clinical nurses.	√	√	
It is recommended to incorporate visual aids in teaching and integrate guidance on managing potential barriers for individuals with NB or caregivers.		√	√
Strengthen instruction for individuals with NB or caregivers on the identification and management of complications.			√
Reinforce shift-to-shift handover protocols and ensure dynamic follow-up guidance throughout the entire CIC management process.		√	
Standardize health education materials and implement personalized education based on patient characteristics to ensure the quality of health education.		√	√
Adopt the “Teach-back” method to evaluate educational outcomes, establishing a closed-loop education model of “education–feedback–re-education.			√
Overcoming the Challenges in Behavioral Practice Transformation and Empowering Self-Management			
A shared decision-making model was adopted to develop a CIC management plan, aiming to enhance adherence among individuals with NB or caregivers.		√	
A structured problem prompt list for CIC self-management was developed to improve the self-management skills of individuals with NB or caregivers.		√	
Digital health tools (WeChat Mini Program) were introduced to strengthen the self-management capabilities of individuals with NB or caregivers.		√	√
Clarifying Diversified Service Needs and Integrating an Optimized Health Support System			
Actively organize patient support groups to leverage the power of peer support in enhancing adherence and treatment confidence.	√	√	
Continuously strengthen the quality of CIC clinical practice guidelines to ensure standardized and evidence-based instructional guidance.		√	
Optimize and diversify health education resources by promoting an integrated “verbal–print–digital” health education model.		√	√
Tertiary hospitals should actively undertake the promotion and training of CIC nursing techniques, optimize the allocation of regional medical resources, and advance the implementation of a coordinated “hospital–community” management model.	√	√	

## 7 Conclusion

This study elucidates two key implementation barriers in the clinical practice of CIC for Chinese individuals with NB or their caregivers: insufficient health literacy and inadequate self-management behaviors. The study also highlights unmet multidimensional needs, including psychological support, social support, and optimized educational resources. Collectively, these findings demonstrate the inadequacy of traditional health education models for CIC management in this context. Therefore, future research should be based on evidence to further explore innovative intervention strategies centered on “empowerment” to overcome the current challenges in CIC management.

## Data Availability

The original contributions presented in the study are included in the article/Supplementary Material, further inquiries can be directed to the corresponding author.
